# Pseudoangiomatous stromal hyperplasia: Presentation and management – a clinical perspective

**DOI:** 10.4102/sajr.v22i2.1366

**Published:** 2018-10-29

**Authors:** Pamela Smilg

**Affiliations:** 1 DRS Incorporated, Johannesburg, South Africa

## Abstract

Pseudoangiomatous stromal hyperplasia (PASH) is a benign breast condition of collagen proliferation. In this article, four cases are presented in series to illustrate the varying clinical presentations of PASH at mammography and sonography, as well as the vastly differing age groups that can be affected. A literature review of the aetiology, pathology and management of PASH is included to provide a comprehensive but succinct overview of the condition, what it is and how to recognise and manage it.

## Introduction

Pseudoangiomatous stromal hyperplasia (PASH) is an uncommon and cryptic breast condition, which was first documented in 1986 when it presented in a patient as a palpable breast mass.^1^ Since then, cases of PASH with varying presentations have been described.^2^

The findings in four different patients imaged at Union Hospital between April 2016 and March 2018 are presented in this report to demonstrate the differing ages and presentations of biopsy-proven PASH. The cases highlight the existence of PASH, its varied forms of presentation and the implications for further patient management.

Although uncommon, it is important to be aware of and recognise this condition in order to avoid submitting the patient to unnecessary surgery or follow-up examinations once it has been diagnosed.

### Case 1

**FIGURE 1:**
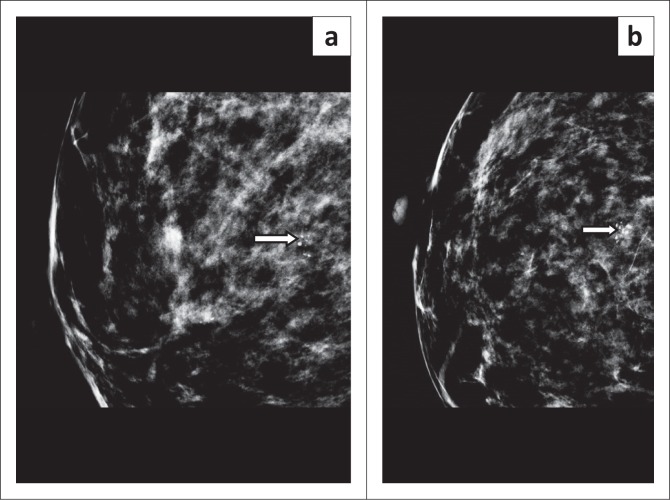
(a) Mediolateral and (b) craniocaudal mammography projections demonstrating clustered indeterminate calcifications in the right breast (arrows).

**FIGURE 2:**
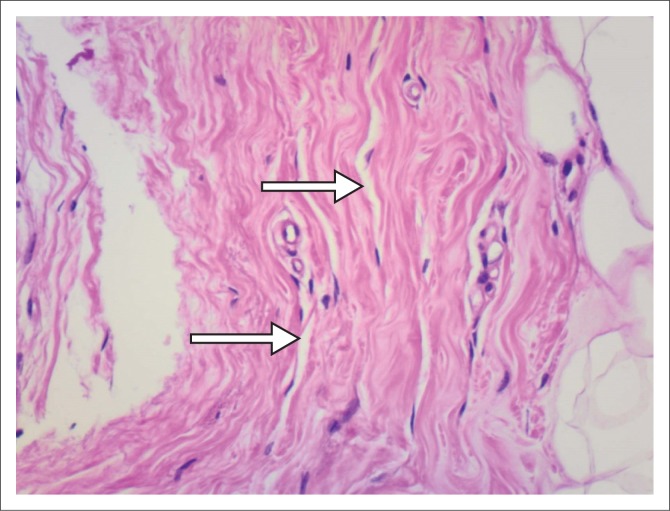
Arrows indicate the pseudovascular spaces.

Miss J, a 40-year-old asymptomatic woman, presented for routine screening. Her mammogram (Figure 1) revealed a cluster of indeterminate microcalcifications in the right breast, which was classified as Breast Imaging Reporting and Data System (BIRADS) IVa, and a stereotactic-guided biopsy was recommended. Breast sonar examination was non-contributory. Pathology demonstrated fibroadenosis with benign microcalcifications and one of the core biopsies showed features of PASH (Figure 2). In this case, PASH was an incidental and unexpected finding.

### Case 2

**FIGURE 3:**
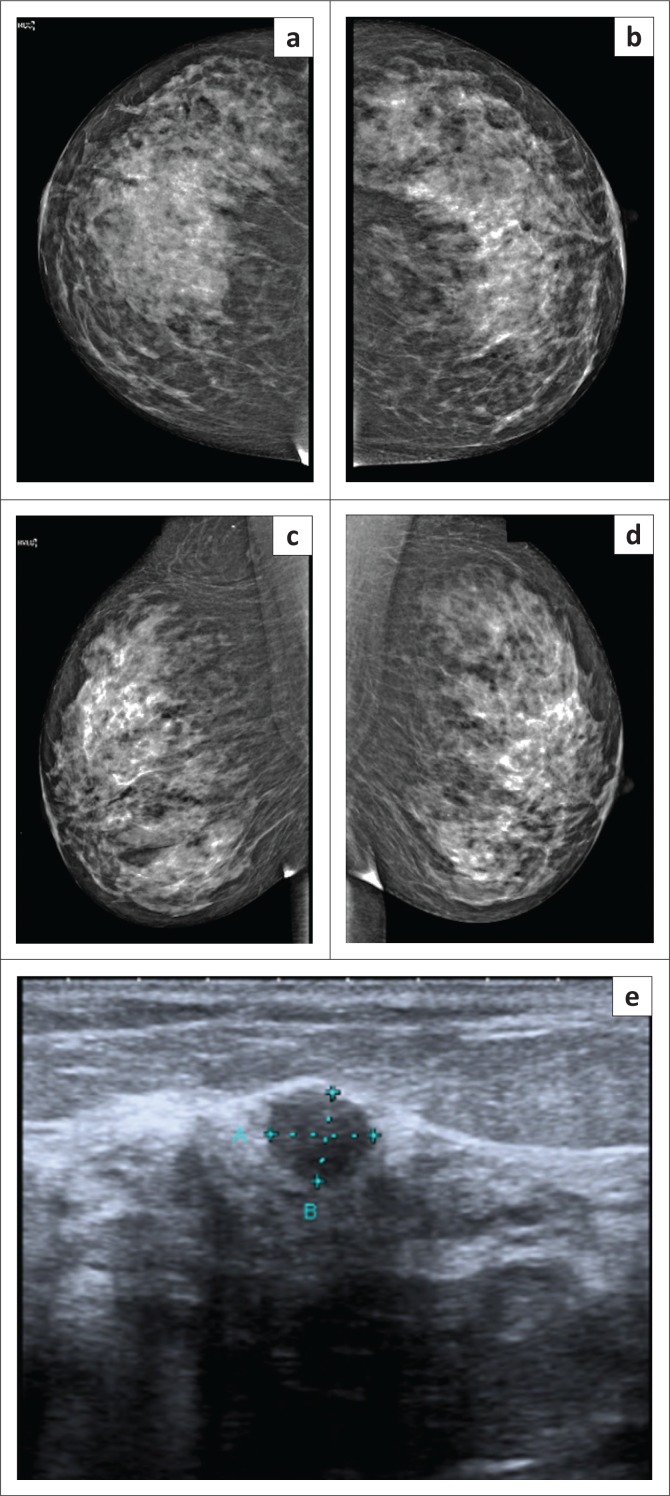
(a–d) Normal appearing craniocaudal and mediolateral mammogram views and (e) right breast sonar image revealing a benign-appearing nodule.

**FIGURE 4:**
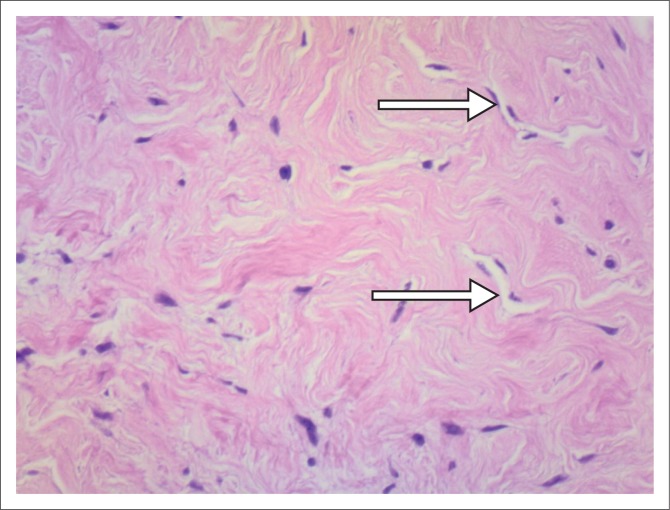
Arrows indicate the pseudovascular spaces.

Mrs K, a 50-year-old asymptomatic woman, was found to have probably benign nodules in her right breast at previous sonography 12 months earlier. At her annual follow-up mammogram and breast sonar, one of these lesions, located superiorly in the right breast, appeared to have increased very slightly in size and become less well defined on sonar (Figure 3e). Her mammogram was non-contributory (Figure 3a–d), and a sonar-guided biopsy was recommended (BIRADS IV). Pathology indicated benign proliferative breast disease with adenosis and PASH (Figure 4).

### Case 3

**FIGURE 5:**
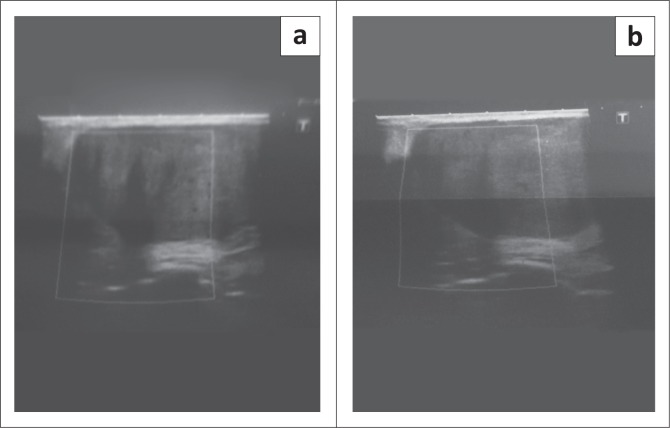
Longitudinal (a) and transverse (b) sonographic images of the predominantly isoechoic, palpable lump.

Miss M, a 14-year-old girl, presented for sonar assessment of a large, growing, palpable lump in her left breast. Corresponding to the lump, there was a 7 cm × 4 cm × 5.7 cm, slightly mixed, predominantly isoechoic, probably benign mass on sonar (Figure 5). Because of its size, sonar-guided core biopsy was recommended (BIRADS IV). Microscopy showed features of PASH.

Immunohistochemical stains were as follows:

- Estrogen receptor (ER) positive in the duct component and negative in the stromal component.
- Progesterone receptor (PR) positive in the epithelial component and negative in the stromal component.
- MNR116 positive in the epithelial component and negative in the stromal component.
- Androgen receptor focal positive in the stromal component.
- CD34 positive in the vascular element only.

### Case 4

**FIGURE 6:**
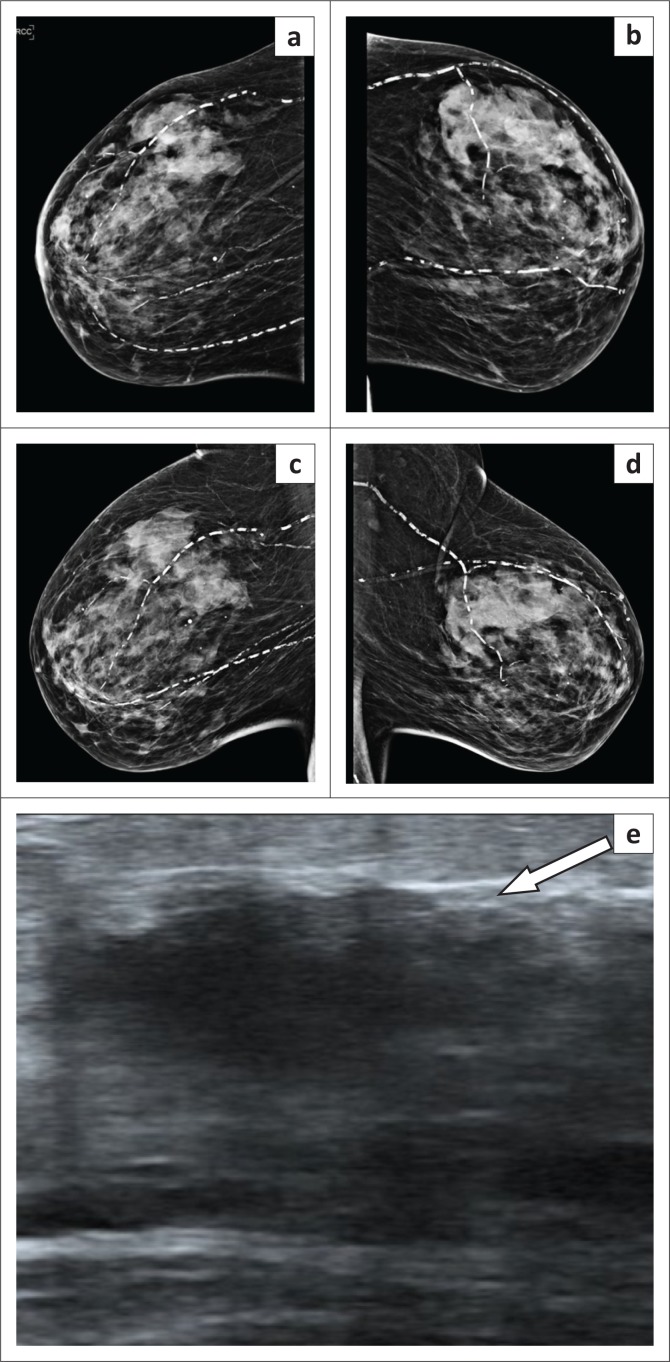
(a–d) Craniocaudal and mediolateral mammogram with a grade D dense breast pattern and (e) sonar demonstrating an area of hypoechogeneity with posterior acoustic shadowing (arrow).

**FIGURE 7:**
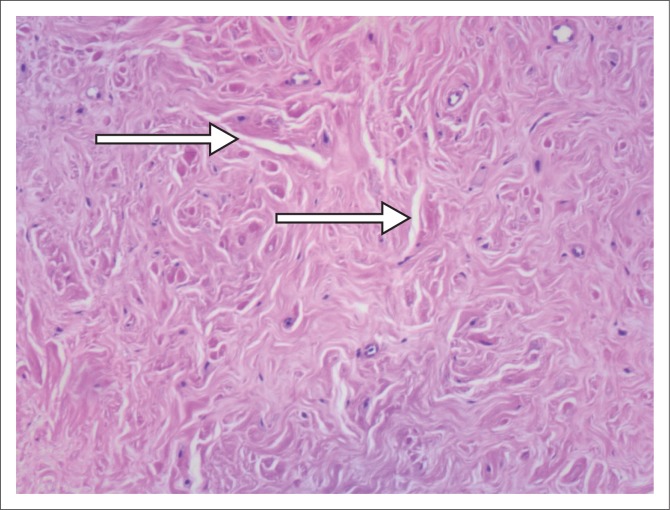
Arrows indicate the pseudovascular spaces.

Mrs Z, a 71-year-old diabetic woman, presented complaining of a palpable lump in the right breast. Her mammogram showed extremely dense tissue with a grade D pattern and increased density in both upper, outer quadrants (Figure 6a). Sonar demonstrated large areas of hypoechogenicity with posterior shadowing bilaterally (Figure 6b), which was difficult to quantify accurately (BIRADS IV). Both diabetic mastopathy and PASH were considered and sonar-guided biopsies of the dominant areas of hypoechogenicity were advised to exclude neoplasia. Pathology indicated features consistent with PASH bilaterally (Figure 7).

## Management and outcome

In all cases, there was radiological and pathological concordance. Annual surveillance was advised for cases 1, 2 and 4. Case 3 required no radiological follow-up but was referred for surgical opinion to discuss the option of surgery in view of possible aesthetic problems because of the size and enlargement of the mass and the patient’s preference. However, the patient did not attend her surgical appointment.

## Discussion

### Aetiology and pathogenesis

Pseudoangiomatous stromal hyperplasia is a benign breast condition of collagen proliferation. The exact aetiology and pathogenesis are unknown, but hormonal factors are known to play a role in the development of PASH; it is more common in pre- and perimenopausal women.^3^ PASH has been shown to occur across a wide age range and has been documented in women from 14 to 67 years old, the majority occurring between 30 and 50 years.^4^ It is rare over the age of 50, which further correlates with a hormonal aetiology.^5,6^ PASH can also occur in men, associated with gynaecomastia.^6^

In known cases of PASH, the lesion size was shown to change with menses, consistent with a hormone-related fluctuation.^1^ In addition, PASH cases have shown strong stromal cell PR positivity and faint stromal nuclear reactivity for oestrogen was shown in one case, whereas the stromal cell nuclei of control cases without PASH did not stain for either receptor.^6^

### Presentation

Clinically, PASH usually presents as a mass, typically enlarging, sometimes rapidly.^4^ It may also present as an incidental, microscopic finding^1,7^ and may or may not be palpable.^2^

Radiologically, the classic description of PASH is of a single, well-circumscribed, round or oval, mobile mass, resembling a fibroadenoma on mammogram and sonar.^5^ On mammography, it typically has the appearance of a probably benign mass,^1^ lacking calcification within it.^2,8^ It varies in size, with measurements of 1 cm–12 cm reported.^8^ On sonar, it is usually a hypoechoic, oval or round, benign-appearing mass but may be slightly heterogeneous.^2,9^ If it occurs within fibroadenosis, the imaging findings reflect this, with mammographically dense tissue and sonographic shadowing, hypoechoic tissue.^9^

On MRI, the findings of PASH are non-specific and range from an enhancing mass to clumped, non-mass-like enhancement, usually with benign kinetics.^2^

However, as illustrated by the above cases, PASH may present with various other findings, none specific to the condition, and indeed, it is most commonly found as an incidental finding in a biopsy specimen, the biopsy having been done for another reason.^9^ It can thus be associated with any other specific pathology or tissue type and the morphology of this may be the dominant finding.

### Pathology

Breast tissue containing PASH exhibits stromal cell proliferation, that is, proliferation of collagen, with slit-like channels lined with myofibroblasts (spindle cells) resembling vascular channels.^5,6^ This is, however, not true angiomatous proliferation and these channels are not blood vessels. Because of this, however, it may be histologically mistaken for a vascular neoplasm.^5^

The spindle cells are positive for Vimentin, CD34, BCL2, CD99 and *α*-smooth muscle actin but negative for CD31 and factor VIII (an endothelium-specific marker). In addition, the cells are hormonally sensitive and frequently express PR and less frequently ER.^1,5,10^

### Differential diagnosis

Radiological differential diagnosis depends on mode of presentation. As a benign-appearing mass, the main differentials are fibroadenoma and phyllodes.^11^ Within an area of fibroadenosis, the condition may be diagnosed as fibroadenosis. PASH may also be misdiagnosed as a different condition at biopsy when it is present as an incidental finding. In addition, PASH may be confused with other diffuse breast diseases such as diabetic mastopathy.^12^

The main pathological differential diagnoses are low-grade angiosarcoma and spindle cell containing entities such as phyllodes and desmoid tumours.

### Management

Pseudoangiomatous stromal hyperplasia is a benign condition. It is neither premalignant nor a risk factor for the development of carcinoma.^6^ It is not associated with synchronous carcinoma^6^ (however, as it occurs together with anything else, it could theoretically coexist as an incidental finding with a carcinoma).

If the imaging findings are equivocal, a histological examination is mandatory for a definitive diagnosis.^6^

A diagnosis of incidental PASH requires no active intervention or radiological follow-up. Pseudoangiomatous stromal hyperplasia is a BIRADS II condition.

Surgery may be performed for enlarging lesions, diffuse PASH with massive breast enlargement which is very rare^12^ and in patients with discordance following triple assessment.^6^ After surgery, it has been shown to have a low rate of recurrence (15% – 22%).^11^

There is no established conservative treatment for PASH. It responds to tamoxifen; however, the effects may only be sustained with prolonged therapy. Long-term tamoxifen may not be ideal because of its side effects^10^ and would be inappropriate in young premenopausal women.

## Ethical considerations

Informed consent was obtained from all the patients for inclusion of their images and information in the study.
